# Classical vs. Customized Post-Traumatic Orbital Reconstruction with ‘In-House’ 3D-Printed Models: An Extended Cohort Study with Volumetric Analysis

**DOI:** 10.3390/jcm15218236

**Published:** 2026-10-22

**Authors:** Elvis Kallaverja, Ida Barca, Francesco Ferragina, Sara Piloni, Antonella Arrotta, Maria Giulia Cristofaro

**Affiliations:** 1 Maxillofacial Surgery Unit, Department of Experimental and Clinical Medicine, Renato Dulbecco Hospital, Magna Graecia University of Catanzaro, 88100 Catanzaro, Italy; 2 Maxillofacial Surgery and Reconstructive Plastic Surgery Unit, Azienda Ospedaliera Ospedali Riuniti Villa Sofia-Cervello, 90146 Palermo, Italy; 3 Department of Medical and Surgical Sciences, Anesthesia and Intensive Care, Magna Graecia University of Catanzaro, Viale Europa, 88100 Catanzaro, Italy

**Keywords:** orbital floor fractures, 3D printing, stereolithographic prototypes, volumetric analysis, patient-specific implants, diplopia

## Abstract

**Background**: Orbital floor fractures (OFFs) represent a challenging reconstructive problem because of the complex three-dimensional anatomy of the orbit. Building upon our group’s 2024 preliminary pilot study, which initially proposed an “In-House” digital framework, this retrospective study aims to definitively validate and scale up that protocol. **Methods**: This retrospective single-center comparative cohort study evaluated an in-house workflow, on patients treated for OFF at the “Magna Graecia” University Hospital of Catanzaro from 1 January 2022 to 31 December 2025, in which titanium mesh was pre-contoured on a patient-specific 3D-printed stereolithographic model, compared with conventional intraoperative manual contouring. **Results**: Twenty-nine patients met the surgical inclusion criteria: 7 were treated conventionally (G1a) and 22 with the 3D-planned workflow (G1b). Twenty-nine patients with intact orbital structures served as a descriptive control cohort (G2). In G1, 87 volumetric measurements were obtained: preoperative measurements of the healthy contralateral and fractured orbits and postoperative measurements of the reconstructed orbit. The primary comparative volumetric outcome was the absolute postoperative residual discrepancy between the reconstructed orbit and the healthy contralateral orbit. Because of the small and unequal groups, the comparison between G1a and G1b was performed using an exact two-sided Mann–Whitney U test. G1b showed a lower median absolute postoperative residual discrepancy than G1a (0.4668 vs. 1.3370 cm^3^; U = 139; *p* = 0.00072). Operative time was also shorter in G1b (mean 41.4 vs. 80.7 min; exact Mann–Whitney *p* < 0.00001). The control cohort showed a mean absolute inter-orbital difference of 0.6177 ± 0.29 cm^3^; the interval 0.3277–0.9077 cm^3^ represents mean ± SD and is reported only as an exploratory descriptive band, not as a validated clinical reference interval or therapeutic threshold. All G1b patients had resolution of diplopia by one year, but the observational design does not establish a causal relationship between a specific volumetric value and functional recovery. **Conclusion**: These findings suggest an association between the in-house 3D workflow and improved volumetric matching and shorter operative time, while the small conventional subgroup and temporal differences between treatment periods require confirmation in larger prospective studies.

## 1. Introduction

In the management of orbital floor fractures (OFFs), the integration of bioengineering allows surgeons to utilize three-dimensional (3D) stereolithographic prototypes and fabricate customized titanium meshes. Based on preoperative computed tomography (CT) data, these technologies aim to improve the accuracy and predictability of orbital floor reduction and reconstruction [1,2].

Despite these technological developments, the literature still lacks standardized recommendations for treating orbital floor fractures. Controversy persists regarding optimal surgical timing, the choice of surgical approach, and the most suitable biomaterials among the numerous options available. Nevertheless, there is general consensus that establishing an appropriate therapeutic strategy requires a detailed clinical examination, a comprehensive orthoptic evaluation, and high-resolution maxillofacial CT imaging [3,4,5,6].

OFFs can impair both facial aesthetics and ocular function. They carry a risk of long-term complications such as diplopia, enophthalmos, infraorbital nerve hypoesthesia, restricted ocular motility, and mechanical lesions that may lead to vision loss. Therefore, the primary objective of surgical management is the morpho-functional rehabilitation of the orbital cavity. With ongoing refinements in diagnostic protocols [2,3] and therapeutic modalities [7,8,9], the quality of the final outcome can be enhanced from both aesthetic and functional perspectives.

In a preliminary study conducted by our group in 2024 [10], we validated the reliability of orbital volume segmentation using the open-source software Horos. That work demonstrated that digital volumetric analysis provides a reproducible method to quantify post-traumatic orbital expansion and predict late sequelae. However, while our baseline study focused on the digital assessment of volume discrepancies, the clinical application of these volumetric data into a routine surgical workflow required further investigation.

To address this issue, our department implemented an “in-house” protocol that utilizes preoperative virtual planning to shape implants on 3D-printed physical models, avoiding standard intraoperative manual adaptation [11,12].

Consequently, the primary objective of the present study is to analyze the variations in orbital volume before and after surgical reconstruction with titanium meshes. This volumetric analysis was conducted via Horos software v4.0.1 on the CT scans of patients with blow-out fractures treated at the Maxillofacial Traumatology Center of the “Magna Grecia” University Hospital of Catanzaro, Italy.

The secondary objective is to evaluate whether utilizing a titanium mesh pre-molded “in-house” on a 3D stereolithographic model provides a more accurate volumetric reconstruction—validated by comparison with the healthy contralateral orbit—than standard preformed titanium meshes bent directly intraoperatively, which are traditionally associated with longer operative times and a higher incidence of postoperative symptoms.

## 2. Materials and Methods

This retrospective comparative study was conducted at the Maxillofacial Traumatology Center of the “Magna Grecia” University Hospital of Catanzaro, Italy.

The study protocol strictly adhered to the Guidelines for Good Clinical Practice (GCP) and the ethical principles outlined in the Declaration of Helsinki. Patient privacy and data confidentiality were maintained in compliance with current national and European data protection regulations. The study received formal approval from the Ethics Committee of the Magna Graecia University of Catanzaro.

The study aims to evaluate two specific endpoints:

**Primary Endpoint:** To quantify and compare pre- and post-operative orbital volume variations in blow-out fracture patients, directly on the patients’ CT scans using OsiriX softwarev14.2.1 This analysis aims to demonstrate how an accurate reconstruction based on the healthy contralateral orbit can effectively restore the original orbital volume, preventing post-operative functional and aesthetic complications (e.g., enophthalmos, diplopia).

**Secondary Endpoint:** To demonstrate that the “in-house” 3D pre-moulding technique provides a more accurate volumetric reconstruction of the orbital cavity compared to the traditional intraoperative moulding method.

**Inclusion and Exclusion Criteria**

Adult patients with a history of orbital trauma and a confirmed diagnosis of an orbital floor “blow-out” fracture were enrolled in this study.

The inclusion criteria were defined as follows:

- Isolated and unilateral orbital floor fracture.
- Preoperative diplopia confirmed by an ophthalmological exam and an orthoptic evaluation using Hess–Lancaster screens.
- Availability of complete CT imaging data.
- Orbital floor reconstruction performed exclusively using titanium mesh.
- Absence of extraocular muscle deficits, nerve and vascular damage.
- Surgical intervention performed within 7 days from the traumatic event
- Surgical procedure executed by the same operating surgeon.

The **exclusion criteria** were defined as follows:

- Patients presenting with concurrent facial skeleton fractures, including zygomaticomaxillary complex (ZMC/COMZ) fractures or panfacial fractures.
- Prior history of surgical treatment for orbital floor fractures or any previous ophthalmic surgery.
- Non-compliant or uncooperative patients.
- Patients with incomplete clinical or radiological documentation.
- Age under 18 years (pediatric population).
- Orbital floor reconstruction performed using materials other than titanium mesh.

The investigation involved a total of 58 patients, who were divided into two main groups to evaluate the clinical efficacy of the different reconstructive techniques (Table 1).

Group 1 (G1) comprised 29 patients presenting with an isolated, unilateral orbital floor “blow-out” fracture treated surgically between 2013 and 2025. To analyze the technological evolution of the materials used, this cohort was further subdivided into two subgroups: G1a, which included 7 patients treated during the earlier years of the study using conventional preformed meshes shaped directly during surgery, and G1b, which consisted of 22 patients treated more recently using titanium meshes pre-contoured prior to surgery on a customized 3D stereolithographic model. Conversely, Group 2 (G2) served as the control group and was made up of 29 patients admitted to the same department for injuries restricted exclusively to the lower third of the face, therefore presenting completely intact orbital structures.

The orbital volume analysis was performed on the patients’ CT scans using OsiriX software, carrying out three precise measurements for each subject in the first group, resulting in a total of 87 volumetric assessments. Specifically, 58 measurements were performed on the preoperative tomographies (29 on the healthy contralateral orbit, taken as the ideal anatomical reference, and 29 on the fractured orbit), while the remaining 29 measurements were conducted on the postoperative CT scans to verify the accuracy of the reconstructed titanium floor.

Similarly, for the 29 patients in the control group (G2), a total of 58 measurements were executed (29 on the left orbit and 29 on the right orbit) with the objective of validating physiological symmetry and establishing standard baseline volumetric parameters. The treatment protocol follows the methodology previously described by Kallaverja et al. in 2024 [6].

### Statistical Analysis

The statistical evaluation of the collected data comprised both descriptive and inferential analyses. Desired parameters were compared using descriptive statistics, reporting continuous variables as means and standard deviations (SD). Baseline clinical and demographic characteristics, including age and sex, were standardized and recorded for all patients across both study cohorts (G1 and G2). For the surgical cohort (G1), additional clinical variables were compiled, including the specific side of the trauma (right or left orbital floor involvement) and the total operative time measured precisely in minutes.

Continuous variables were summarized using means and standard deviations and, where appropriate for the small samples, medians. For each patient in G1, the postoperative residual volumetric discrepancy was defined as the difference between the postoperative reconstructed orbital volume and the healthy contralateral orbital volume; for comparative analysis, its absolute value was used. This variable corresponds to the manuscript’s ‘Recovery Difference’ and directly quantifies residual postoperative mismatch with the contralateral reference orbit. It was compared between G1a and G1b using an exact two-sided Mann–Whitney U test because the groups were small and unequal in size. Operative time was analyzed similarly as an unadjusted secondary outcome. The Bravais–Pearson correlation between healthy contralateral and postoperative reconstructed orbital volumes was retained only as a secondary descriptive analysis of linear association and was not interpreted as a measure of accuracy, agreement, treatment superiority, or causal efficacy. Given the retrospective design and the different treatment periods, all between-group analyses were considered unadjusted and potentially confounded. No causal inference was attempted.

- The preoperative volume difference between the healthy contralateral orbit and the fractured orbit.
- The volume difference within the injured orbit between its preoperative and postoperative states.
- The absolute difference between the postoperative volume of the reconstructed orbit and the baseline volume of the healthy contralateral orbit, which served as the primary comparative metric to evaluate the efficacy of the patient-specific, 3D-planned pre-contoured mesh versus intraoperative shaping.

Tomographic images, acquired with bone reconstruction filters and a 0.5 mm slice thickness, were processed using RadiAnt software v2025.2 [13].

To ensure reproducibility, multiplanar reconstruction (MPR) alignment along standardized anatomical axes and subsequent slice-by-slice volumetric segmentation of the orbital cavities were performed using Horos software, following the validation protocol previously described by our group [10]. The orbital volume was calculated using a hybrid manual and semi-automated mode, utilizing open- and closed-polygon tools to manage bony deficiencies and herniated tissues within the blow-out fractures.

For patient-specific reconstruction, preoperative DICOM datasets were processed to generate stereolithography (STL) files for 3D printing. The virtual mesh was optimized, sliced, and printed as a physical thermoplastic polyurethane (TPU) anatomical model. The titanium mesh was then mechanically adapted and contoured directly onto this 3D-printed model to establish stable support points, restore anatomical fidelity, and safeguard vital neurovascular structures [10].

## 3. Results

The descriptive analysis provides a comprehensive overview of the collected datasets. Within this framework, the volumetric means establish the central tendency of the samples, while the corresponding standard deviations quantify the statistical dispersion of the individual measurements around these central values.

### 3.1. Surgical Cohort Analysis G1

**Figure 1:**
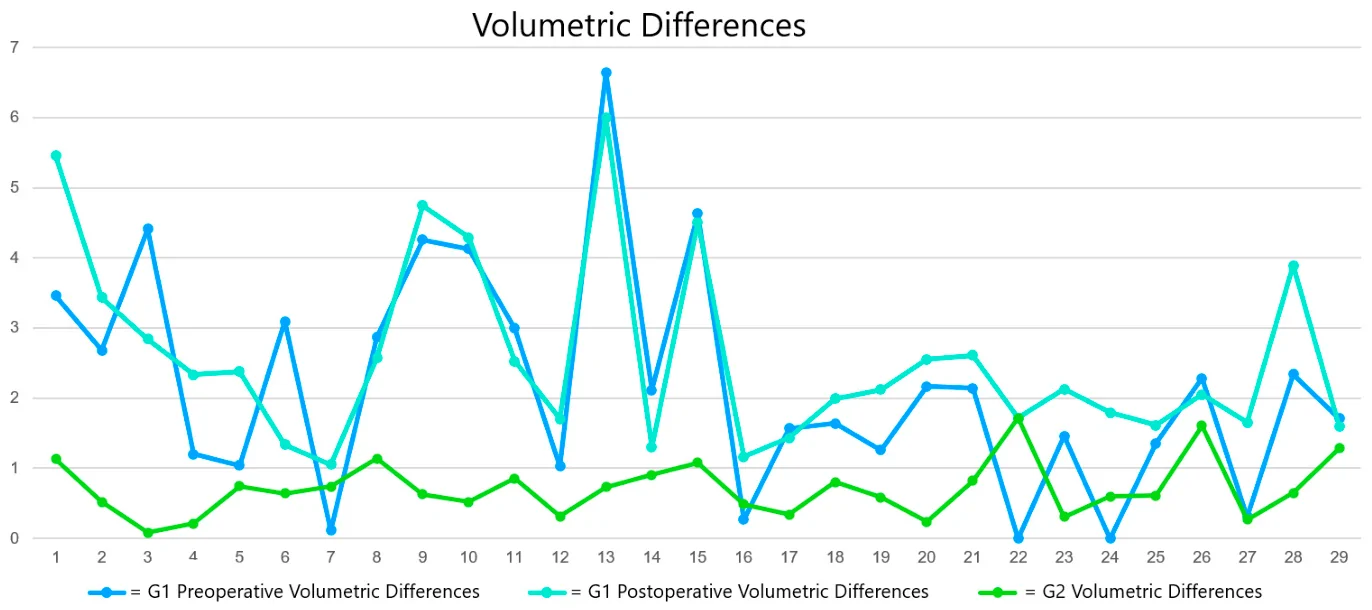
Comparison of the differences in preoperative and postoperative volumes in G1 with volumetric differences in G2. (G1 pre = blue, G1 post = light blue, G2 = green).

The descriptive statistics for the surgical cohort (G1), which includes patients presenting with an isolated, unilateral orbital floor “blow-out” fracture, provide a comprehensive overview of the demographic data and volumetric variations (Table 1 and Table 2, Figure 1):

G1 comprises 29 patients with orbital fractures (20 males and 9 females) with a mean age of 51 ± 19.1 years.

Orbital volume analysis revealed a mean right orbital volume of 23.97 cm^3^ (standard deviation, SD = 3.02), corresponding to 24.6763 cm^3^ (SD = 3.05) in males and 22.4005 cm^3^ (SD = 2.43) in females. For the left orbit, the mean volume was 24.0769 cm^3^ (SD = 2.92), averaging 24.6567 cm^3^ (SD = 2.97) in males and 22.7885 cm^3^ (SD = 2.49) in females.

Analysis of the inter-orbital volume discrepancy demonstrated a mean difference of 2.9779 cm^3^ (SD = 1.71). Specifically, the mean discrepancy was 2.9012 cm^3^ (SD = 1.33) in males and 3.2847 cm^3^ (SD = 3.27) in females.

The mean volume variation in the fractured orbit between the preoperative and postoperative phases was 3.099 cm^3^ (SD = 1.57), averaging 3.1744 cm^3^ (SD = 1.30) in males and 2.7972 cm^3^ (SD = 2.78) in females.

### 3.2. Surgical Cohort Analysis G2 (Table 3, Figure 1)

G2 comprises 29 healthy patients (22 males and 7 females) with a mean age of 44.1 ± 14.43 years.

Orbital volume analysis revealed a mean right orbital volume of 23.9147 cm^3^ (standard deviation, SD = 1.99), corresponding to 24.5371 cm^3^ (SD = 1.83) in males and 21.9588 cm^3^ (SD = 0.94) in females. For the left orbit, the mean volume was 23.971 cm^3^ (SD = 2.28), averaging 24.6258 cm^3^ (SD = 2.02) in males and 21.9129 cm^3^ (SD = 1.86) in females.

Analysis of the inter-orbital volume discrepancy demonstrated a mean difference of 0.6177 cm^3^ (SD = 0.29). Specifically, the mean discrepancy was 0.6185 cm^3^ (SD = 0.30) in males and 0.6136 cm^3^ (SD = 0.26) in females.

### 3.3. Inferential Statistics and Correlation Analysis

For the primary comparative volumetric outcome, the absolute postoperative residual discrepancy was lower in G1b than in G1a. In G1a (n = 7), the mean absolute residual discrepancy was 1.3560 ± 0.4474 cm^3^ and the median was 1.3370 cm^3^. In G1b (n = 22), the corresponding values were 0.5634 ± 0.4376 cm^3^ and 0.4668 cm^3^. The exact two-sided Mann–Whitney U test showed a statistically significant unadjusted difference between groups (U = 139, *p* = 0.00072). This result indicates that the 3D-planned group had smaller postoperative residual volumetric discrepancies in this cohort, but it does not establish causal superiority because the groups were treated in different periods and may differ with respect to unmeasured temporal or clinical factors.

The control cohort had a mean absolute inter-orbital difference of 0.6177 ± 0.29 cm^3^. The interval 0.3277–0.9077 cm^3^ therefore corresponds to mean ± SD rather than the observed range. The actual observed control values ranged from 0.0828 to 1.7137 cm^3^. For descriptive purposes only, 1/7 patients in G1a (14.3%) and 11/22 patients in G1b (50.0%) had absolute postoperative residual discrepancies within the mean ± SD band of the control cohort.

The Bravais–Pearson linear correlation coefficient (r) was calculated to evaluate the relationship between the baseline healthy orbit and the final volume of the surgically reconstructed cavity. Normality testing confirmed that both the healthy and postoperative reconstructed orbital volumes followed a normal distribution (Table 3). The analysis demonstrated a strong, statistically significant linear correlation between the healthy contralateral side and the treated orbit, indicating that the surgical reconstruction effectively re-established a volumetric configuration that closely matches the patient’s native anatomy.

**Figure 2:**
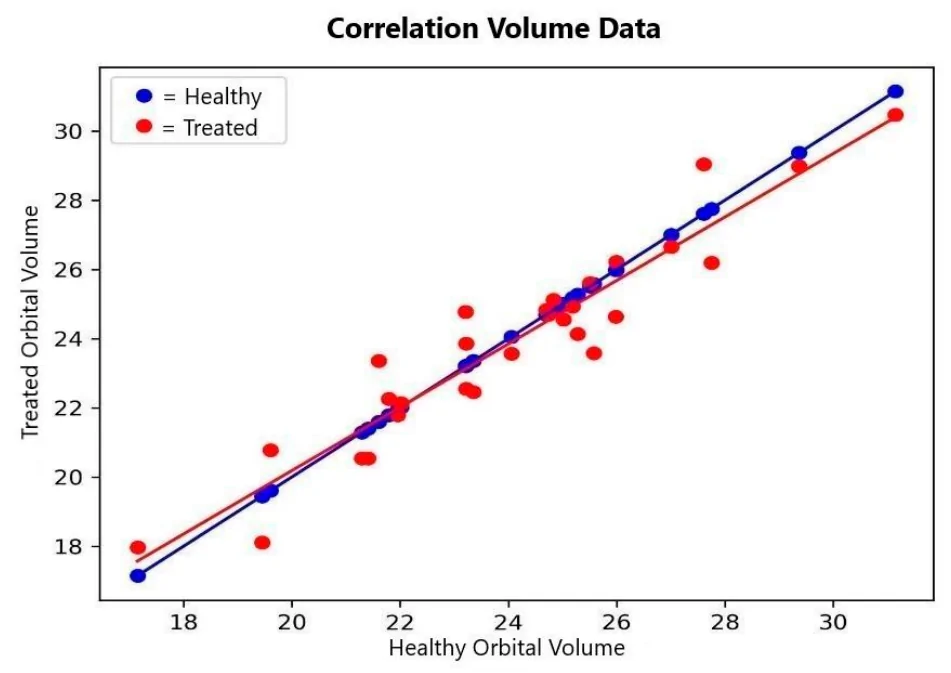
Graph showing the linear relationship between healthy orbital volumes and treated orbital volumes.

The Bravais–Pearson correlation analysis demonstrated a linear relationship, with a correlation coefficient of r = 0.95 (*p* < 0.0001). To evaluate the precision and stability of this correlation, a 95% confidence interval (CI) was calculated using Fisher’s Z-transformation, yielding a narrow range between 0.896 and 0.977 (Figure 2).

## 4. Discussion

### 4.1. Anatomical Challenges and the Evolution of Reconstruction

The complex three-dimensional anatomy of the orbit—characterized by delicate bony walls, specific landmarks such as the posteromedial bulge, and close relationships with vital neurovascular and ocular structures—makes orbital floor fractures (OFFs) a significant challenge in maxillofacial traumatology [7]. Over the last decade, the primary objective of surgical intervention has shifted from simple mechanical closure of the defect to rigorous morpho-functional rehabilitation of the orbital cavity. Historically, traditional reconstructive methods relied heavily on the surgeon’s free-hand, intraoperative intuition to shape titanium meshes or autologous bone grafts. This empirical approach introduced a high degree of unpredictability, often resulting in suboptimal volumetric restoration and long-term functional sequelae, including persistent diplopia, enophthalmos, or restricted ocular motility [14,15].

The introduction of point-of-care 3D bioengineering and virtual surgical planning (VSP) has altered this landscape, transferring digital precision directly to the operating room via “in-house” stereolithographic models. However, despite these technological developments, contemporary international scientific literature still lacks standardized, globally accepted guidelines regarding optimal surgical timing, approach, and biomaterial selection [16,17].

### 4.2. Validation of the Volumetric Framework and Clinical Relevance

The present data support the feasibility of quantitative postoperative orbital volumetry and suggest an association between the in-house 3D-planned workflow and closer volumetric matching to the healthy contralateral orbit. However, the statistical interpretation requires caution. A strong correlation between contralateral and postoperative orbital volumes indicates that larger native orbits tend to be associated with larger reconstructed orbits, but correlation does not measure agreement or surgical accuracy and cannot demonstrate treatment superiority. Accordingly, the Pearson correlation should be interpreted only as a secondary descriptive finding.

The direct comparison of absolute postoperative residual discrepancies provides a more clinically interpretable measure of volumetric mismatch. In this cohort, G1b showed lower absolute residual discrepancies than G1a, with an exact Mann–Whitney *p*-value of 0.00072. Nevertheless, this is an unadjusted retrospective comparison between groups treated in different periods. The observed difference may reflect the 3D workflow, but it may also reflect secular changes in surgeon experience, patient selection, perioperative care, imaging, or other unmeasured factors. Therefore, the result should be considered hypothesis-generating rather than definitive evidence of causal superiority.

### 4.3. Interpretation of the Control Band and Diplopia

The control cohort demonstrated a mean absolute inter-orbital discrepancy of 0.6177 ± 0.29 cm^3^. The interval 0.3277–0.9077 cm^3^ should not be described as a physiological reference interval because it was derived from mean ± SD in only 29 controls and was not externally validated. In addition, the observed control values extended from 0.0828 to 1.7137 cm^3^. We therefore use the term ‘exploratory descriptive band’ and do not propose it as a therapeutic target.

The clinical findings also do not establish a direct causal relationship between a specific volumetric discrepancy and diplopia resolution. Although all G1b patients were free of diplopia at one year, only 11 of 22 patients had absolute postoperative residual discrepancies within the exploratory band. Thus, the present data cannot demonstrate that remaining within that band is necessary or sufficient for functional recovery. Orbital volume is one component of postoperative ocular function, which may also be influenced by muscle injury, soft-tissue edema, fibrosis, preoperative displacement, nerve function, and other factors. Prospective studies with standardized ophthalmologic and orthoptic endpoints are needed to determine whether a quantitative volumetric target has independent prognostic value.

### 4.4. Comparison of Surgical Techniques

**Figure 3:**
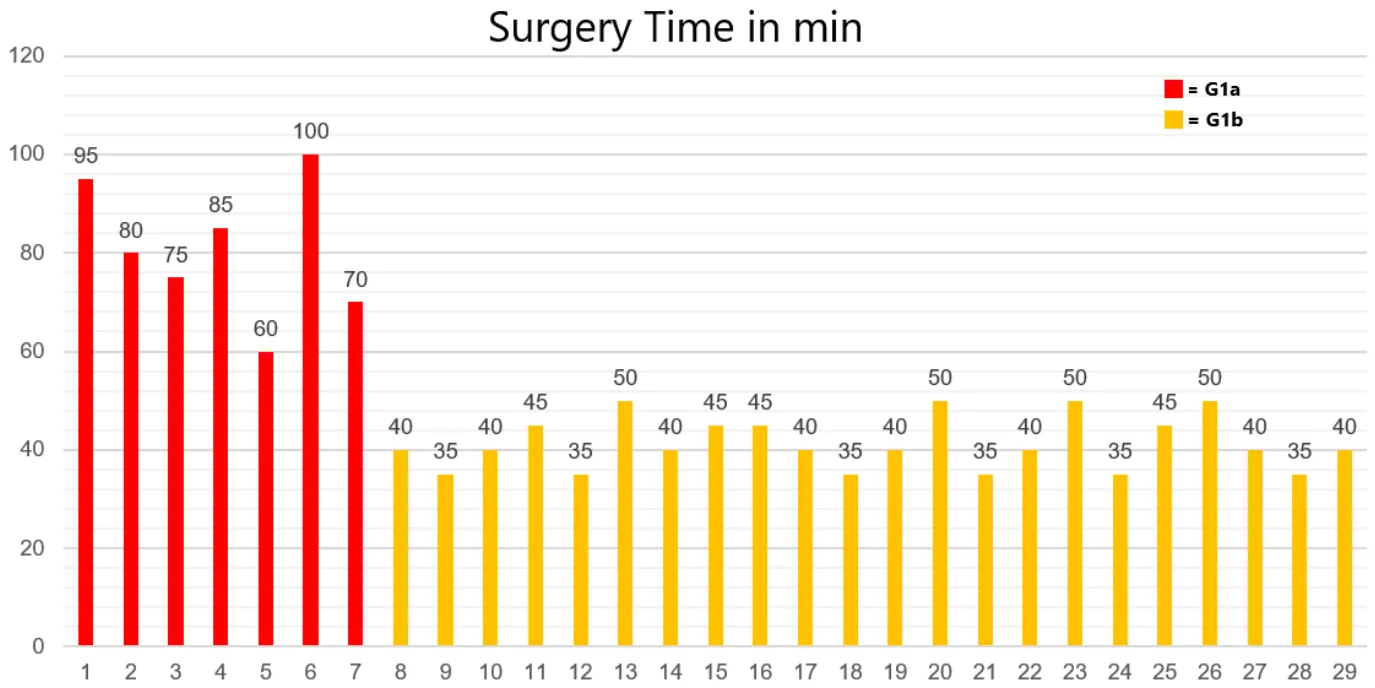
Comparison of surgical timing between G1a and G1b.

The shorter operative time observed in G1b is consistent with the practical rationale for preoperative mesh contouring. The mean operative time was 80.7 ± 14.0 min in G1a and 41.4 ± 5.4 min in G1b. The exact Mann–Whitney comparison was statistically significant; however, this finding is also vulnerable to temporal and learning-curve effects because the conventional and 3D-planned procedures were performed in different periods. The 40-min difference should therefore be interpreted as an observed workflow difference in this cohort rather than as a definitive causal estimate of time saved by 3D planning (Figure 3).

### 4.5. The Technical Superiority of the “In-House” Pre-Planned Workflow

When comparing the two surgical methodologies, distinct clinical and geometric differences emerged between G1a (traditional intraoperative manual contouring) and G1b (“in-house” preoperative 3D-planned modeling). In G1b, which comprised the majority of the surgical sample (22 patients), all subjects achieved complete resolution of diplopia by the 1-year follow-up; this included two patients who experienced transient, delayed recovery that resolved fully by the 6th postoperative month. In contrast, all patients in G1a (7 patients) exhibited either delayed healing or permanent functional deficits, including two individuals with persistent diplopia one year after surgery.

The volumetric data presented in Table 2 and Table 3 provide a pathophysiological explanation for these differences. In G1a, the absolute volume discrepancies deviated substantially from the physiological reference standard, showing marked variations characterized by either excessive or insufficient corrections. Notably, the two patients with persistent diplopia at one year represented the extreme outliers of this manual cohort, exhibiting the largest volumetric errors. One patient presented a severe over-correction (−1.9963 cm^3^), which restricted the orbital cavity volume and induced postoperative exophthalmos due to soft-tissue compression; the other experienced an under-correction of 1.7595 cm^3^.

These complications are often inherent to free-hand intraoperative bending. Contouring a rigid titanium mesh within a restricted, obscured surgical field through a transconjunctival or subciliary approach forces the clinician to rely on subjective visual estimation and repeated adaptations. This process risks misplacing the implant relative to the stable posterior bony landmarks and can cause progressive metal fatigue, compromising the structural integrity of the mesh. Conversely, the “in-house” 3D pre-molding protocol allows the surgeon to adapt the mesh preoperatively onto an exact stereolithographic model of the patient’s skeleton. This approach ensures the implant aligns with the healthy contralateral anatomy, avoids intraoperative positioning errors, and places the mesh precisely onto stable anatomical margins to maintain the orbit within physiological volume thresholds.

### 4.6. Surgical Efficiency, Anesthesia Exposure, and Workflow Optimization

From a clinical and patient-management standpoint, this 40-min optimization offers several benefits. First, minimizing the duration of anesthesia exposure correlates with a reduction in systemic postoperative complications, smoother extubation phases, and decreased cognitive impact, particularly in elderly trauma patients. Second, shortening the open wound time reduces the risk of intraoperative bacterial contamination and subsequent surgical site infections or retrobulbar implant complications. Finally, from an institutional perspective, reducing surgical times optimizes operating room turnover, enhances clinical efficiency, and lowers hospital costs.

While commercial patient-specific implants (PSIs) fabricated by external vendors can offer comparable precision, they often introduce logistical delays, requiring several days for manufacturing and shipping, and generate substantial financial costs that may burden public healthcare systems. Our point-of-care, “in-house” workflow addresses these limitations. By utilizing desktop 3D printers within the department, custom stereolithographic models can be fabricated within hours, providing a precise, rapid, and cost-effective solution compatible with the urgent timelines of maxillofacial trauma management.

### 4.7. Validation and Longitudinal Progress Relative to Preliminary Data

The clinical and statistical findings of the present study consolidate and expand the preliminary trends originally reported by our group in 2024 [10]. In our pilot series, which evaluated a limited cohort of 10 surgical patients (5 in G1a and 5 in G1b) and 10 controls, we hypothesized that an “in-house” 3D-planned preoperative workflow could offer advantages over traditional manual shaping. The current study, expanded to a clinical sample of 29 surgical patients, provides further validation of that initial hypothesis.

**Figure 4:**
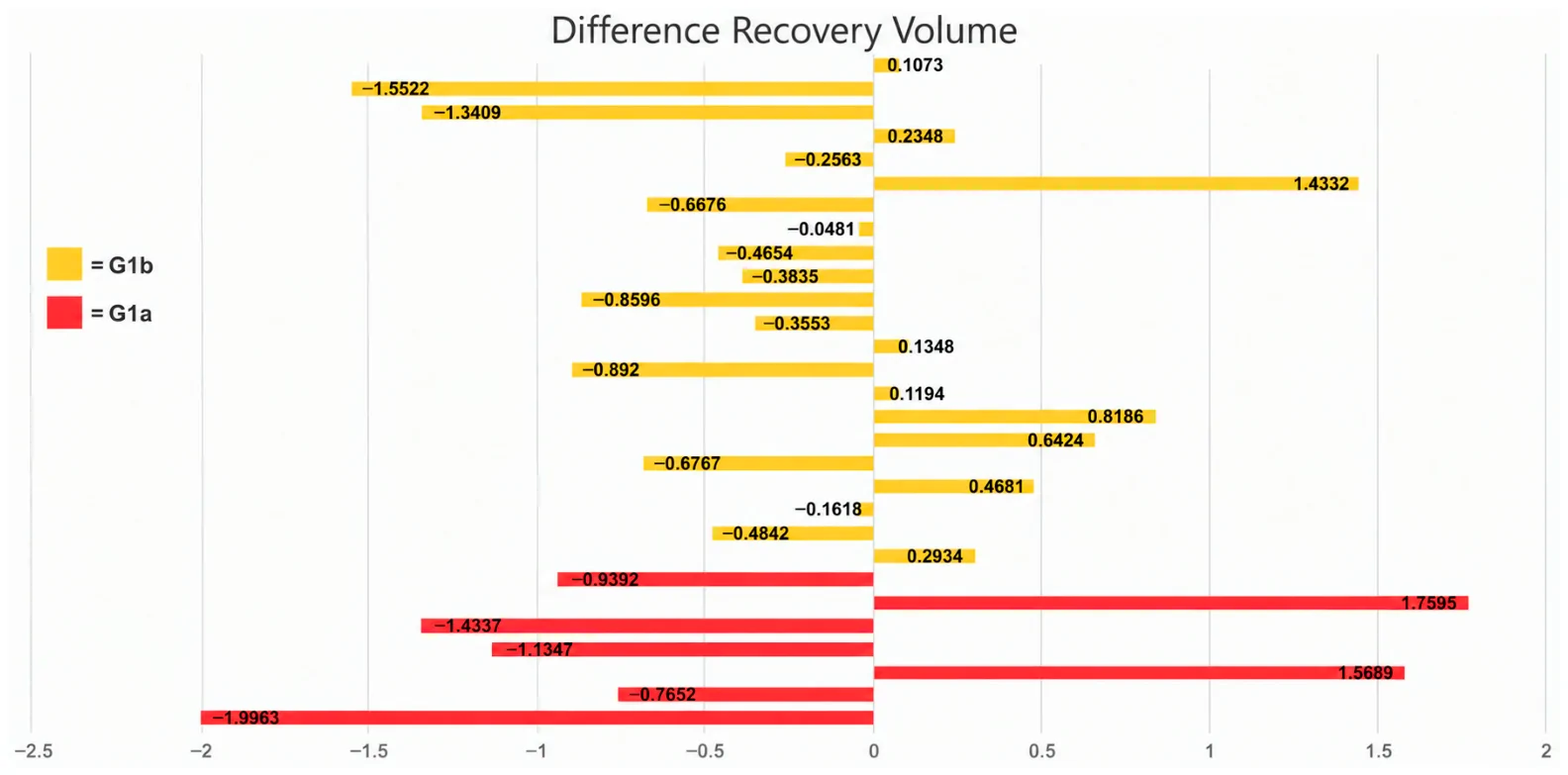
Difference Recovery Volume.

A central aspect of this research line is the progressive reinforcement of our predictive models. Our baseline 2024 Bravais–Pearson correlation coefficient of r = 0.9350 aligned with the current r = 0.95 (*p* < 0.0001), which is backed by a narrow 95% confidence interval (0.896–0.977). This consistency demonstrates that the digital protocol maintains high volumetric precision when scaled to a larger patient population. Furthermore, the physiological inter-orbital baseline discrepancy range established in our current control cohort (mean ± SD of G2) remains consistent with the mean value identified in our 2024 report (0.6351 cm^3^) (Figure 4).

This consistency across different patient samples firmly establishes this specific volumetric window as a reproducible, standardizable therapeutic target for orbital floor reconstruction (Table 4).

When analyzing the pathological outliers, our current Group 1a findings align with and reinforce the negative trends observed in our early series. The severe over- and under-corrections observed in the traditional group, which directly caused long-term functional impairment, confirm that manual free-hand bending does not guarantee predictable volumetric outcomes. Notably, the operational efficiency of the digital workflow demonstrated consistent stability over time. Pre-modeling the mesh preoperatively on 3D-printed stereolithographic models consistently achieved an intraoperative time saving of 40 min, reproducing the timeline optimization recorded in our 2024 baseline data. Consequently, this expanded study marks the transition of the “in-house” 3D-planned protocol from an innovative protocol to a validated, reliable clinical reference for the management of orbital floor fractures.

## 5. Conclusions

In this retrospective single-center cohort, the in-house 3D-planned preoperative mesh-contouring workflow was associated with a smaller postoperative residual volumetric discrepancy from the healthy contralateral orbit and a shorter operative time than conventional intraoperative mesh contouring. These findings support the feasibility and potential clinical utility of the workflow but do not establish causal superiority, universal reproducibility, or a validated therapeutic volumetric threshold. The conventional subgroup was small (n = 7), treatment periods differed, and residual confounding cannot be excluded. The proposed 0.3277–0.9077 cm^3^ interval represents mean ± SD in the control cohort and should not be considered a validated physiological or therapeutic range. Likewise, the observed resolution of diplopia cannot be attributed causally to achieving a particular orbital volume. Larger prospective, preferably multicenter, comparative studies with standardized functional outcomes are required before the workflow can be considered a clinical standard.

## 6. Limitations

The retrospective single-center design introduces potential selection bias and limits control of confounding variables. The conventional subgroup comprised only seven patients, resulting in substantial imbalance relative to the 22 patients treated with the 3D workflow and limiting the precision of subgroup estimates. The two techniques were used during different treatment periods; consequently, differences may reflect temporal changes in surgeon experience, perioperative management, patient selection, or other factors rather than the 3D workflow alone. The present dataset does not permit adequate adjustment for these potential confounders, and the between-group results should therefore be interpreted as observational associations [10,18,19,20].

## Figures and Tables

**Table 1:** Data of patients in G1a (treated with intra-op-shaped titanium mesh).

	Age	Sex	Fracture Side	Volume cm^3^ Right Orbit	Volume cm^3^ Left Orbit	Volume cm^3^ Mesh Orbit	Vol-cm^3^ Difference Pre-op	Vol-cm^3^ Difference Post-op	Vol-cm^3^ Recovery Difference	Surgery Time (min)
**1**	33	M	Right	29.0359	25.5777	23.5814	3.4582	5.4545	−1.9963	95
**2**	59	M	Right	23.9741	21.2927	20.5365	2.6814	3.4376	−0.7562	80
**3**	37	M	Left	23.204	27.6138	24.7729	4.4098	2.8409	1.5689	75
**4**	74	M	Left	25.2797	26.4807	24.145	1.201	2.3357	−1.1347	85
**5**	41	M	Left	25.9815	27.0248	24.6445	1.0433	2.3803	−1.337	60
**6**	66	F	Left	21.6021	24.6969	23.3616	3.0948	1.3353	1.7595	100
**7**	46	F	Right	19.7235	19.6073	20.7789	0.1162	1.0554	−0.9392	70

**Table 2:** Data of patients in G1b (treated with preop-shaped titanium mesh).

	Age	Sex	Fracture Side	Volume cm^3^ Right Orbit	Volume cm^3^ Left Orbit	Volume cm^3^ Mesh Orbit	Vol-cm^3^ Difference Pre-op	Vol-cm^3^ Difference Post-op	Vol-cm^3^ Recovery Difference	Surgery Time (min)
**1**	19	Male	Right	27.6954	24.8297	25.1231	2.8657	2.5723	0.2934	40
**2**	72	Male	Left	24.0521	28.3124	23.5679	4.2603	4.7445	−0.4842	35
**3**	37	Male	Left	21.9546	26.0827	21.7928	4.1281	4.2899	−0.1618	40
**4**	30	Male	Left	21.7904	24.7852	22.2585	2.9948	2.5267	0.4681	45
**5**	19	Male	Left	31.149	32.1745	30.4723	1.0255	1.7022	−0.6767	35
**6**	73	Female	Left	23.2221	29.8653	23.8645	6.6432	6.0008	0.6424	50
**7**	62	Male	Right	19.2656	17.1499	17.9685	2.1157	1.2971	0.8186	40
**8**	42	Male	Left	22.0159	26.6465	22.1353	4.6306	4.5112	0.1194	45
**9**	78	Female	Right	23.6237	23.354	22.462	0.2697	1.1617	−0.892	45
**10**	73	Female	Right	26.2649	24.6941	24.8289	1.5708	1.436	0.1348	40
**11**	63	Female	Right	28.6397	27.0017	26.6464	1.638	1.9933	−0.3553	35
**12**	69	Female	Left	21.4052	22.6647	20.5456	1.2595	2.1191	−0.8596	40
**13**	53	Male	Right	31.5383	29.37	28.9865	2.1683	2.5518	−0.3835	50
**14**	73	Male	Left	25.0174	27.16	24.552	2.1426	2.608	−0.4654	35
**15**	27	Male	Left	24.7357	26.403	24.6876	1.6673	1.7154	−0.0481	40
**16**	48	Female	Right	24.6782	23.2198	22.5522	1.4584	2.126	−0.6676	50
**17**	23	Male	Right	30.8342	27.609	29.0422	3.2252	1.792	1.4332	35
**18**	61	Male	Left	25.1795	26.5356	24.9232	1.3561	1.6124	−0.2563	45
**19**	24	Male	Right	28.273	25.9876	26.2224	2.2854	2.0506	0.2348	50
**20**	51	Female	Right	19.7573	19.448	18.1071	0.3093	1.6502	−1.3409	40
**21**	75	Male	Right	30.0882	27.7497	26.1975	2.3385	3.8907	−1.5522	35
**22**	51	Male	Left	25.5081	27.2179	25.6154	1.7098	1.6025	0.1073	40

**Table 3:** Data of patients in G2.

	Age	Sex	Volume cm^3^ Right Orbit	Volume cm^3^ Left Orbit	Vol-cm^3^ Difference
**1**	23	Male	24.3835	25.5133	1.1298
**2**	55	Male	24.2495	24.7679	0.5184
**3**	39	Male	24.4433	24.5261	0.0828
**4**	47	Male	20.3659	20.5763	0.2104
**5**	53	Male	23.5792	22.8334	0.7458
**6**	28	Male	22.7471	23.386	0.6389
**7**	19	Male	27.9404	27.2011	0.7393
**8**	38	Male	23.0517	21.9158	1.1359
**9**	46	Male	23.5677	24.1943	0.6266
**10**	57	Male	28.6717	29.1944	0.5227
**11**	63	Female	23.1834	24.0362	0.8528
**12**	54	Female	22.6238	22.9362	0.3124
**13**	37	Male	25.7352	26.4701	0.7349
**14**	41	Male	24.8725	23.9635	0.909
**15**	67	Male	24.5537	25.636	1.0823
**16**	75	Female	20.6917	20.2034	0.4883
**17**	25	Male	25.8289	26.1691	0.3402
**18**	56	Female	20.8196	20.0188	0.8008
**19**	34	Male	25.1794	25.7635	0.5841
**20**	42	Male	27.1861	26.9465	0.2396
**21**	29	Male	23.6145	24.4392	0.8247
**22**	60	Female	22.5522	24.2659	1.7137
**23**	58	Female	22.1071	21.8021	0.305
**24**	37	Male	24.6925	24.094	0.5985
**25**	22	Male	23.5787	22.9672	0.6115
**26**	54	Female	21.734	20.1278	1.6062
**27**	46	Male	24.8968	25.1679	0.2711
**28**	33	Male	24.1362	24.7853	0.649
**29**	41	Male	22.5412	21.257	1.2842

**Table 4:** Comparative parameter.

Comparative Parameter	Preliminary Pilot Study (2024)	Present Expanded Study (2026)
**Surgical Sample Size (G1)**	N = 10 patients (5 G1a/5 G1b)	N = 29 patients (7 G1a/22 G1b)
**Bravais–Pearson Correlation (r)**	r = 0.9350 (*p* < 0.0001)	r = 0.9500 (*p* < 0.0001, 95% CI: 0.896–0.977)
**Physiological Discrepancy (G2)**	Mean ± SD: ± 0.6351 cm^3^	Range: 0.3277–0.9077 cm^3^
**1-Year Diplopia Resolution (G1b)**	Complete resolution observed	50% of patients free from diplopia
**Surgical Time (G1a vs. G1b)**	79 min vs. 39 min (Δ 40 min)	81 min vs. 41 min (Δ 40 min)
**Workflow Status**	Methodological Pilot Project	Clinical Reference

## Data Availability

The original contributions presented in this study are included in the article. Further inquiries can be directed to the corresponding author(s).
